# Targeting different types of human meningioma and glioma cells using a novel adenoviral vector expressing GFP-TRAIL fusion protein from hTERT promoter

**DOI:** 10.1186/1475-2867-11-35

**Published:** 2011-10-28

**Authors:** Jessica T Li, Ka Bian, Alan L Zhang, Dong H Kim, William W Ashley, Rahul Nath, Ian McCutcheon, Bingliang Fang, Ferid Murad

**Affiliations:** 1 Department of Neurosurgery, The University of Texas Medical School at Houston, 6400 Fannin Street, Houston, TX 77030, USA; 2 Department of Thoracic and Cardiovascular Surgery, The University of Texas M. D. Anderson Cancer Center, Houston, Texas 77030, USA; 3 Department of Neurosurgery, The University of Texas M. D. Anderson Cancer Center, Houston, Texas 77030, USA; 4 Texas Nerve and Paralysis Institute, 6400. Fannin St., Houston, Texas 77030, USA; 5 Department of Biochemistry and Molecular Biology, The George Washington University Medical Center, 2300 I Street, NW, Washington, DC 20037, USA

## Abstract

**Objective:**

The objective of this study was to evaluate the anti-tumor effects of Ad/gTRAIL (an adenoviral vector in which expression of GFP and TRAIL is driven by a human telomerase reverse transcriptase promoter, hTERT) on malignant meningiomas and gliomas.

**Background:**

Gliomas and meningiomas are the two most common types of human brain tumors. Currently there is no effective cure for recurrent malignant meningiomas or for gliomas. Ad/gTRAIL has been shown to be effective in killing selected lung, colon and breast cancer cells, but there have been no studies reporting its antitumor effects on malignant meningiomas. Therefore, we tested the antitumor effect of Ad/gTRAIL for the first time in human malignant meningioma and glioma cell lines, and in intracranial M6 and U87 xenografts.

**Methods:**

Materials and Methods: Human malignant meningioma and glioma cells were infected with adenoviruses, Ad/gTRAIL and Ad/CMV-GFP. Cell viability was determined by proliferation assay. FACS analysis and quantification of TRAIL were used to measure apoptosis in these cells. We injected Ad/gTRAIL viruses in intracranial M6 and U87 xenografts, and measured the brain tumor volume, quantified apoptosis by TUNEL assay in the brain tumor tissue.

**Results:**

Our studies demonstrate that in vitro/in vivo treatment with Ad/gTRAIL virus resulted in significant increase of TRAIL activity, and elicited a greater tumor cell apoptosis in malignant brain tumor cells as compared to treatment with the control, Ad/CMV-GFP virus without TRAIL activity.

**Conclusions:**

We showed for the first time that adenovirus Ad/gTRAIL had significant antitumor effects against high grade malignant meningiomas as well as gliomas. Although more work needs to be done, our data suggests that Ad/gTRAIL has the potential to be useful as a tool against malignant brain tumors.

## Background

Gliomas and meningiomas are the two most common types of human brain tumors. Malignant gliomas are the most aggressive and deadliest type of brain tumor[1]. Meningiomas, on the other hand, are usually benign but often recur after surgical removal. They can undergo malignant transformation, and depending on their location can be serious and even potentially lethal to patients [2]. It has been reported that there has been a steady increase in the incidence of malignant brain tumors in both adults and children [3]. There is no effective long-term treatment for this disease. Cellular and molecular therapies including novel vector based gene therapy are currently being studied in preclinical and clinical settings for intracranial malignancies [4-6].

Telomerase is the cellular enzyme responsible for the replication of chromosomal ends or telomeres. It is a multiunit ribonucleoprotein complex that contains an essential RNA component, human telomerase RNA (hTR) and essential protein components including the rate-limiting catalytic subunit human telomerase reverse transcriptase (hTERT). The strong link between telomerase activity and cancer was initially reported by Kim et al [7]. Using a highly sensitive PCR based telemetric repeat amplification protocol (TRAP) assay, they detected telomerase activity in many advanced tumors but not in normal somatic tissues or benign tumors. Since then, all major types of cancer have been screened for telomerase activity. It has been estimated that more than 85% of human cancers have high telomerase activity, which makes telomerase the most common tumor marker. Not only has telomerase been proposed as a diagnostic and prognostic marker for cancer, telomerase inhibition has been widely tested as a potential anticancer strategy [8]. In addition, the high tumor-specificity of hTERT gene expression and the fact that hTERT expression is mainly regulated at the transcriptional level have prompted the use of an hTERT promoter to drive suicide genes to induce specific cancer cell killing using liposome or adenovirus delivery systems [9].

TRAIL, a member of the TNF family, triggers apoptosis through interactions with death receptors (DR4 and DR5) on the cell surface. We and others have shown that direct transfer of the full-length coding sequence of the human tumor necrosis factor-related apoptosis-inducing ligand (TRAIL) into cancer cells elicited apoptosis and apoptotic bystander effects on malignant cells and suppressed tumor growth *in vivo*. More recently, we constructed a bicistronic adenoviral vector expressing the GFP-TRAIL fusion protein from the hTERT promoter via GAL4 gene regulatory system (Ad/gTRAIL) and demonstrated that Ad/gTRAIL treatment effectively elicited apoptosis in various tumor cells *in vitro *and suppressed xenograft tumor growth *in vivo*, with no detectable toxicity in human primary hepatocytes [10]. Since the hTERT promoter is tumor specific, expressing the TRAIL gene by hTERT could overcome the possible liver toxicity reported for TRAIL gene expression.

In this report, we determined the expression level of hTERT in benign and malignant meningioma and glioma cells and investigated the expression and efficacy of Ad/gTRAIL in these cells. Our data showed that Ad/gTRAIL is effective against highly malignant meningioma and gliomas without toxicity to normal cells in the brain and suggest that Ad/gTRAIL may have potential usage in malignant brain tumor therapy.

## Results and Discussion

### hTERT expression level in primary cultured meninglioma cells and glioma cell lines

**Figure 1:**
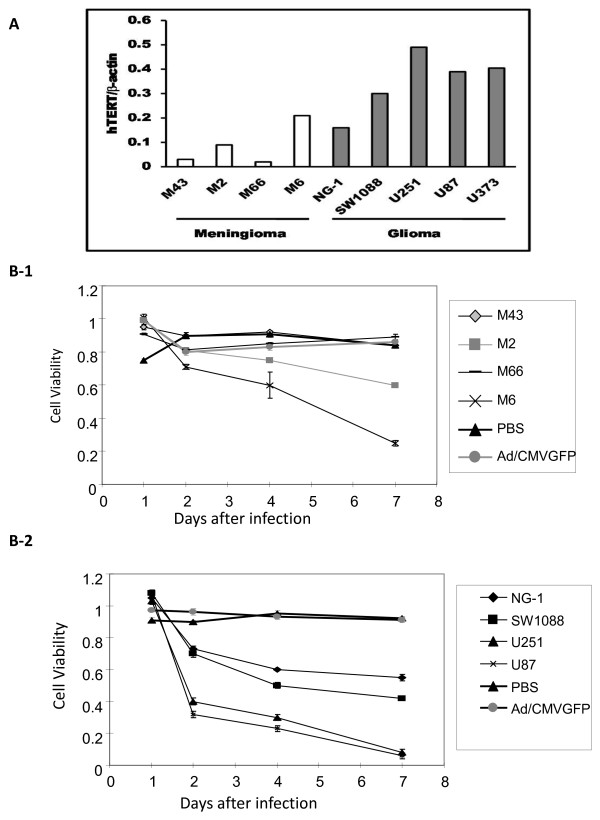
**Endogenous hTERT activity and cell killing effect of Ad/gTRAIL**: (A) Endogenous hTERT mRNA level in meningioma and glioma cells. The hTERT mRNA level in primary cultured meningioma cells and glioma cell lines was determined by real time quantitative PCR as described the Methods. The levels of β-actin was used as endogenous controls. (B) In vitro cell-killing effect of Ad/TRAIL on various brain tumor cells. (B-1) Meningioma cells (B-2) Glioma cells. Cell viability was determined by XTT assay. Cells treated with PBS were used as a control, and their viability was set at 1. Values are mean ± SD for two quadruplicate assays PBS (mock control), Ad/CMV-GFP (vector control), and M43, M2, M66, M6 and NG-1 SW1088, U251, U87, Ad/gTRAIL., Cells were treated with MOI of 1000 VPs/ cell at 7 consecutive days. Cell viabiligy of M6, U251, and U87 cells treated with Ad/gTRAIL was significantly different (p< 0.001) from that of Ad/CMV-GFP and PBS treated cells.

In general, hTERT expression levels correlate with the degree of malignancy in cancer cells. We evaluated the hTERT mRNA expression in primary cultured meningioma cells and glioma cell lines using real-time RT Q-PCR technique. As shown in (Figure 1A), very low levels of hTERT expression were detected in three benign meningioma cells (M43, M2, and M66) while higher level hTERT was noted in M6 malignant meningioma cells. All 3 higher grade glioma cell lines (U251, U87, and U373) had significantly elevated hTERT expression. However, NG-1 cells, derived from surgical specimens of human gliomas (established in the Department of Neuro-Oncology at The University of Texas M. D. Anderson Cancer Center) showed a lower expression of hTERT[11]. The low grade SW1088 glioma cell line also demonstrated decreased hTERT levels [12]. These results confirmed that hTERT mRNA expression correlates with the malignancy level of brain tumor cells.

### Correlation between GFP-TRAIL expression and hTERT level

**Figure 2:**
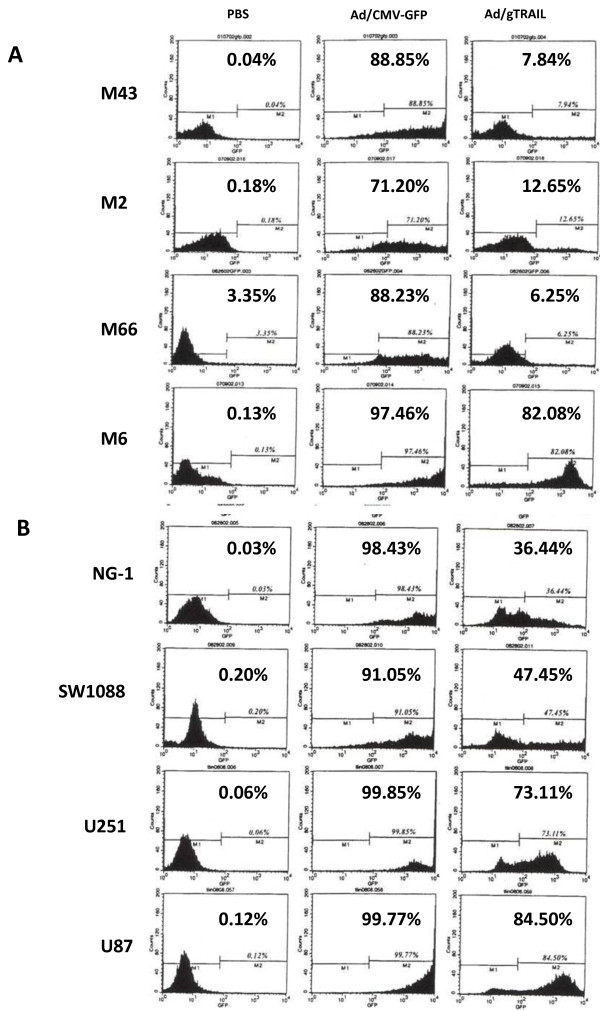
**Expression of GFP-TRAIL in brain tumor cells**: (A) Meningioma cells and (B) Glioma cells. The GFP-TRAIL expression after treatment with Ad/gTRAIL was determined by flow cytometric analysis. Cells treated with PBS or Ad/CMV-GFP was used as negative or positive control. On the left side, cell lines; at the top, treatments; and percentages of cells expressing GFP are shown in each panel.

hTERT is over expressed in up to 90% of cancer cells[13,14]. Moreover, Ad/gTRAIL has been shown to be selectively active in cancer cells. To test the effect of Ad/gTRAIL on malignant brain tumors, we treated meningioma cells (benign: M43, M2, M66; malignant: M6) and glioma cells (NG-1, SW1088, U251, U87) with Ad/gTRAIL or Ad/CMV-GFP. Cells treated with PBS were used as a control. Cells were harvested 48 hr after virus treatment, and then divided into two parts. One part was used for GFP expression detection, and another part was used for apoptosis analysis. As shown in (Figure 2A), benign meningioma cells (M43, M2, M66) treated with Ad/gTRAIL resulted in similar lower levels of GFP-positive cells (7.94%, 12.65% and 6.25% respectively), suggesting that hTERT promoter activity was minimal in these cells. On the other hand, Ad/gTRAIL infected M6 malignant meningioma cells demonstrated significantly higher GFP-positive cells (82.08%), reflecting increased hTERT promoter activity. Similarly, the two highly malignant glioma cell lines (U251 and U87) also had a high percentage of GFP-positive cells (73.1% and 84.5% respectively), indicating high hTERT promoter activity (Figure 2B). The cells lines with intermediate hTERT expression (NG-1 and SW1088) showed 36.4% and 47.4% GFP-positive cells respectively (Figure 2B). Cells with PBS treatment have no significant GFP-positive cells detected. There was no significant difference in GFP-positive cells among different group of cells treated with control vector Ad/CMV-GFP.

### Targeting TRAIL induced apoptosis in malignant cells by Ad/gTRAIL

**Figure 3:**
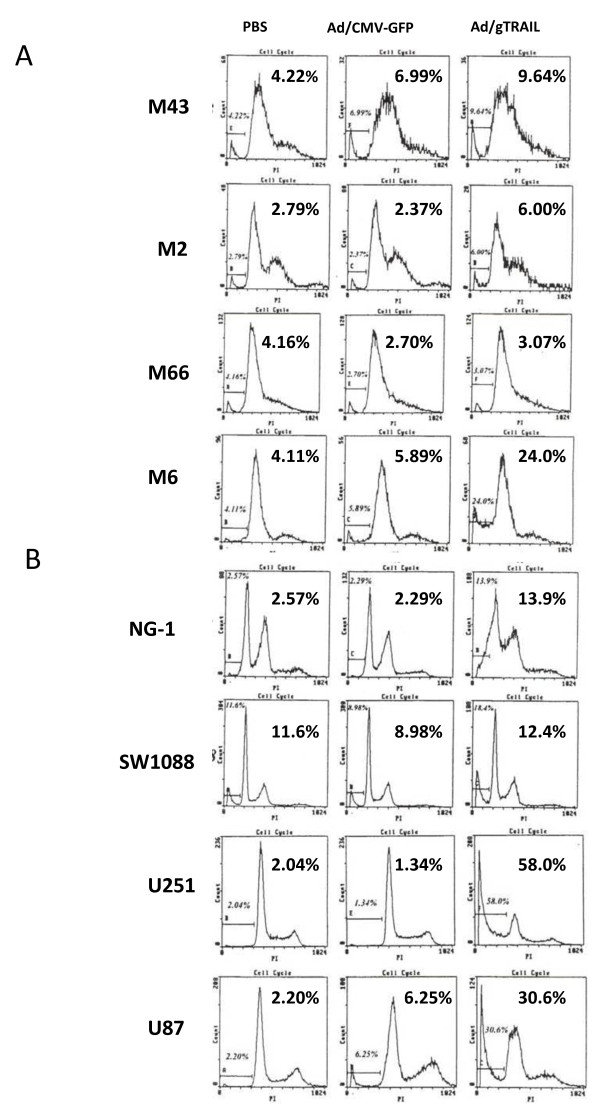
**Induction of apoptosis by Ad/gTRAIL**: (A) Meningioma cells and (B) glioma cells were treated with PBS, Ad/CMV-GFP or Ad/gTRAIL at MOI of 1000 VPs/ cell. Apoptosis were determined by flow cytometric assay at 4 days after the treatment. On the left side, the name of cell lines; at the top, treatments; and percentages of apoptosis are shown.

Treatment of benign meningioma cells (M43, M2, M66) with Ad/gTRAIL did not change the apoptosis rate when compared to cells treated with control vector Ad/CMV-GFP, indicating lack of hTERT-targeted mechanism. However, malignant meningioma M6 cells treated with Ad/gTRAIL showed 24% apoptosis rate, which is 4-fold higher than cells treated with control Ad/CMV-GFP (Figure 3A). In addition, Ad/gTRAIL induced significant apoptosis (58% and 30.6%) in highly malignant U251 and U87 cells respectively (Figure 3B). The percentages of apoptosis for intermediate level hTERT expressing cell lines are: NG-1, 13.9% and SW1088, 18.4% (Figure 3B). Cells with PBS treatment have no effect on apoptosis induction. It is noteworthy that control vector Ad/CMV-GFP did not induce additional apoptosis as to PBS treated groups.

### Cytopathic effect of Ad/gTRAIL on human brain tumor cells

**Figure 4:**
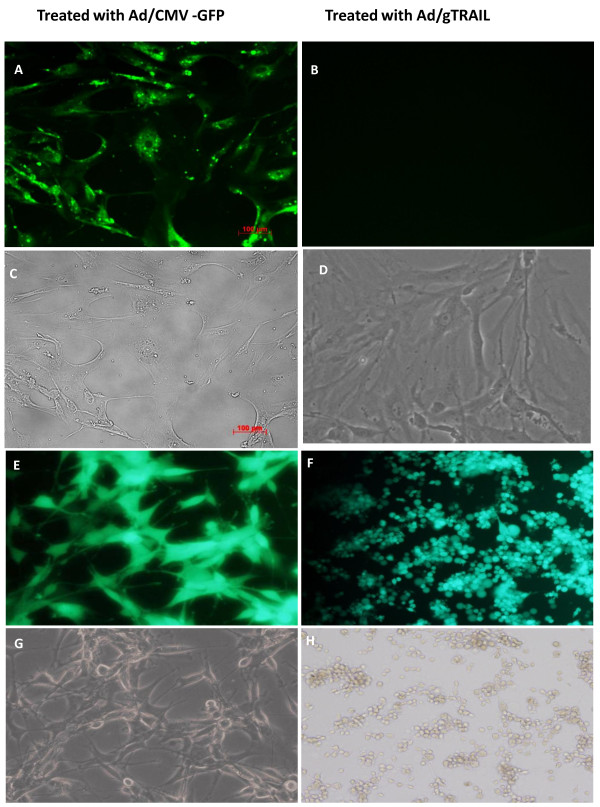
**In vitro gene expression and cell killing**: (A - D) Meninglioma cells (M66) treated with Ad/CMV-GFP (A, C) or Ad/gTRAIL (B, D). Benign meningioma cells were treated with Ad/CMV-GFP or Ad/gTRAIL at 1000 MOI Expression of GFP or its fusion protein was revealed under a fluorescent microscope at 5 days after the treatment. (E-H) Glioma cells (U87) treated with Ad/CMV-GFP (E, G) or Ad/gTRAIL (F, H). Malignant Glioma cells treated with Ad/CMV-GFP or Ad/gTRAIL as described for meninglioma cells. Expressions of GFP or its fusion protein (A, B, E, F) were revealed under a fluorescent microscope. The cells morphologies (C, D, G, H) were revealed under regular microscope. Note, glioma cells treated with Ad/gTRAIL showed apoptosis morphology with the membrane blebbing and cell shrinkage and apoptosis.

To determine whether the infection of human brain tumor cells with either Ad/gTRAIL or Ad/CMV-GFP would have a cytopathic effect, we performed dose-dependence assays with low-hTERT benign meningioma M66 cell, and high-hTERT glioma U87 cell. The cells were seeded at 1 × 10^4 ^cells/well in flat-bottom 12-well plates. After an overnight incubation, cells were divided into 3 groups and treated with PBS, Ad/CMV-GFP, Ad/g-TRAIL respectively. The treatment concentration of Ad/CMV-GFP, Ad/g TRAIL were at MOIs of 500, 1000 and 3000 VPs/ cell. Benign meningioma cells were evenly infected by the control vector Ad/CMV-GFP, showing strong green fluorescence (Figure 4A).

However, due to selective expression of GFP-TRAI, green fluorescence was observed only in malignant cells (Figure 4F), and not in benign cells, (Figure 4B). This suggests that after Adv/g TRAIL infection, differential expression of the adenoviral transgene is mediated by the tumor-selective promoter system and not influenced by cellular susceptibility to virus infection. As shown in (Figure 4C &4D), no obvious morphologic changes were observed up to 5 days after all the treatments, suggesting that even at an MOI of 3000 VPs/cell, Ad/gTRAIL is not toxic to, and has no cytopathic effect on benign meningioma cells. In comparison, obvious apoptotic morphologic changes were observed in the maligant glioma cells after treatment with Ad/gTRAIL at an MOI of 1000 VPs/cell (Figure 4F &4H). This result was consistent with our previous observation that transgene expression from the hTERT promoter after adenovector-mediated gene delivery is high in malignant cells but minimal in normal cells [10,15]. This result also demonstrated that hTERT promoter activity was in different levels in different grade brain tumor cells.

### Anti-proliferation effect of Ad/gTRAIL on human brain tumor cells

To obtain further evidence that the hTERT promoter can drive tumor specific expression of the TRAIL gene in different grade menigioma and glioma cells, we used the XTT assay to compare cell viability after treatment with Ad/gTRAIL in all meningioma (Figure 1.B-1) and glioma cells (Figure 1.B-2). The cell viability was significantly lower in Ad/gTRAIL treatedU251, U87-glioma cells (p< 0.001), and M6 malignant meningioma cells (p<0.001) when compared to the control Ad/CMV-GFP and PBS treated. The increased cell death in U87 and U251 glioma, and M6 malignant meningioma cells was directly correlated with hTERT expression level (Figure 3).

### Apoptosis pathway elicited by Ad/gTRAIL in human brain tumor cells

**Figure 5:**
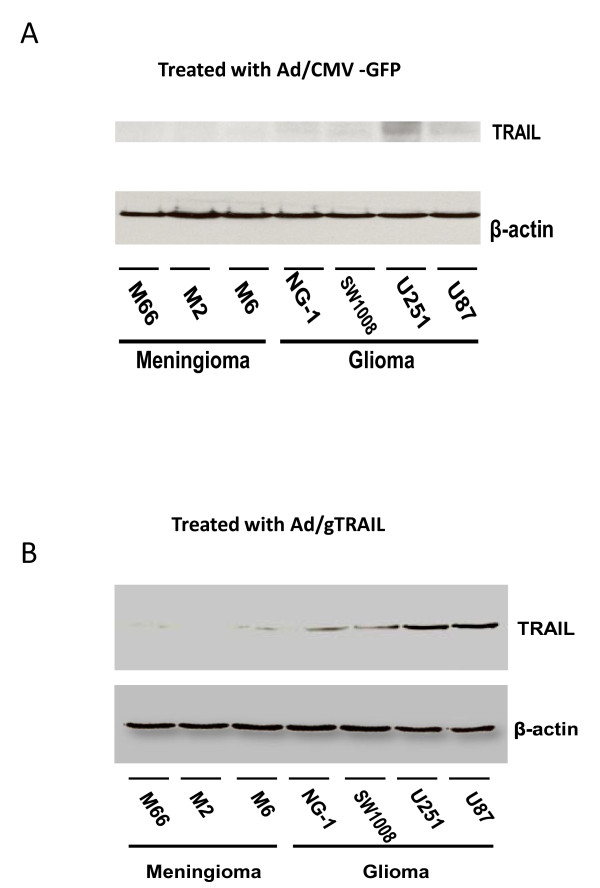
**The TRAIL gene expression in brain tumor cells**: (A) Cells were treated with Ad/CMV-GFP at 1000 MOI VPs/ cell at 3 days. (B) Cells were treated with Ad/gTRAIL at 1000 MOI VPs/ cell at 3 days. Protein levels were analyzed by Western blot analysis after the treatment. β-actin was used as loading control.

The induction of apoptosis by Ad/gTRAIL was confirmed by western blot analysis (Figure 5). The TRAIL expression was observed in cells treated with1000 MOI Ad/CMV-GFP at 3 days (A),. and treated with 1000 MOI Ad/gTRAI at 3 days (B). However, there was an increased expression of TRAIL in meninglioma M6 and glioma U251 and U87 cells treated with Ad/gTRAIL when compared to benign cells. Additionally, our data demonstrated that Ad/gTRAIL was more effective in causing cell death and apoptosis induction in malignant human brain tumor cells, but not in benign tumor cells. This result suggests that apoptosis is involved in cell death produced by the TRAIL gene therapy.

### Therapeutic effect of Ad/gTRAIL in intracranial xenografted M6 and U87

**Figure 6:**
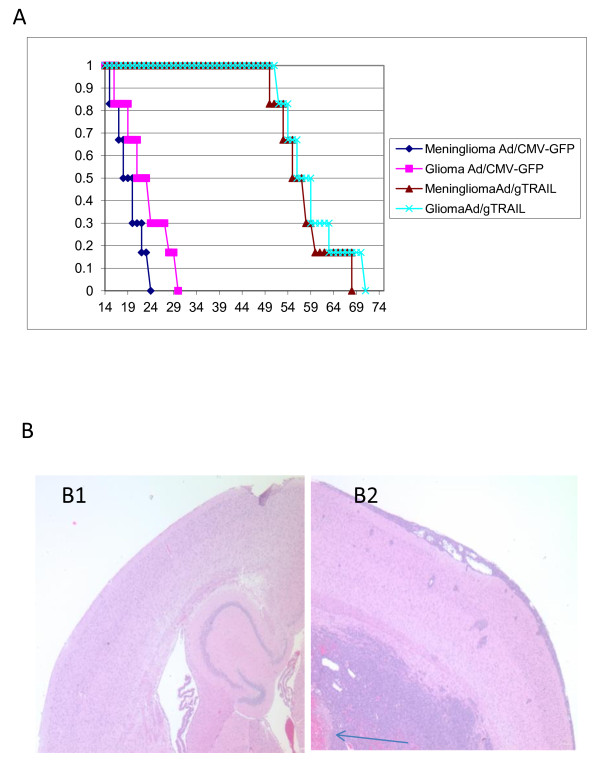
**In vivo antitumor activity of Ad/gTRAIL**: (A) Survival of animals treated with Ad/gTRAIL. Nude mice bearing intracranial M6 and U-87 xenografts were injected intratumorally with Ad/CMV-GFP or Ad/gTRAIL at doses of 1.5 × 10^8 ^viral particles in 5 μL for 3 days. The Kaplan-Meier survival curve for each group was shown in the paragraph (n = 6 animals in each group). The mean survival for the control mice Ad/CMV-GFP) was 19 days in M6 and 26 days in U87 respectively. In contrast, the survival for the Ad/gTRAIL treated mice was 61 days in M6 and 63 days in U87, which was statistically significantly longer than the mean survival of control mice (p< 0.05). (B) The athymic mice brain section (B1) with Meninglioma xenograft (B2).

To evaluate the therapeutic effect of Ad/gTRAIL in brain tumors *in vivo*, we measured the anti-tumor growth efficacy of Ad/gTRAIL in intracranial xenografted M6 meningioma and U87 glioma, and compared it with that of control vector Ad/CMV-GFP, and. In four independent experiments, the mean survival for the control mice (mice receiving Ad/CMV-GFP) was 19 days (95% CI = 14 to 24) in meningioma, and 22 days (95% CI = 15 to 30 days) in glioma respectively. In contrast, the survival for the Ad/gTRAIL treated mice was 61 days (95% CI = 50 to 69 days) in meningioma, and 63 days (95% CI = 52 to 71 days) in glioma which was statistically significantly longer than the mean survival of the control mice (p< 0,05; log-rank test), (Figure 6A).

### Histopatholgic Examination of Tumor in Brain

Microscopic examination of the brain of control mice with M6 meningioma xenografts revealed non-infiltrative tumor growing in a spherical pattern (Figure 6B1). Histological characteristics of the tumors included a dense cellular mass, and the tumor center had necrotic areas (Figure 6-B2 Arrow). All the brains of M6 and U-87 xenografted mice that died naturally showed a midline shift and ventricular compression secondary to tumor-mass effects, which are characteristic features of herniation, and indicate that growth was probably the cause of death in mice that died naturally.

### Immunohistochemical staining for TUNEL assay

**Figure 7:**
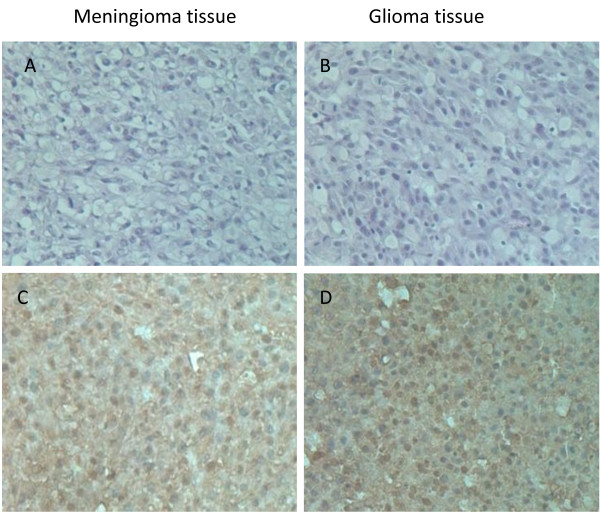
**In vivo assess apoptosis by TUNEL assay**: Tissue section of meningioma (A, C) and glioma (B, D) at 53 days after treatment. (A) meningioma and (B) glioma treated with Ad/CMV-GFP. (C) meningioma and (D) glioma treated with Ad/gTRAIL. TUNEL staining was performed as described in the Methods.

To assess apoptosis induction in vivo, we performed terminal deoxynucleotidyl transferase-mediated dUTP labeling (TUNEL) staining on tumor sections in 2 treatment groups. Brown color indicates apoptotic nuclei as visualized using the DAB substrate. Apoptosis was calculated as percentage of at least 1000 second cells. As shown in (Figure 7), treatment Ad/gTRAIL in meninglioma resulted in significantly higher apoptotic 63.2% (C), when compared to control Ad/CMV-GFP 4.4% (A). In glioma, treatment Ad/gTRAIL resulted in higher apoptotic index 68.9% (D), compared to Ad/CMV-GFP 5.2% (B). Result of in situ TUNEL staining in tumor meningioma and glioma section.

## Conclusions

The tumor necrosis factor related apoptosis-inducing ligand (TRAIL) has received a great attention in cancer treatment because it induces apoptosis in cancer cells but not in normal cells. TRAIL belongs to a small subset of proapoptotic protein ligands in the TNF superfamily [16,17], which triggers apoptosis through interactions with death/TRAIL receptors (DR4/TRAIL-R1 and DR5/ TRAIL-R2)[18], and activation of caspase-8 at the death-inducing signaling complex (DISC) on the cell surface[19]. TRAIL-receptor agonists are considered as promising cancer therapeutics, and are currently under evaluation in early clinical trials[20]. Recombinant human Apo2L/TRAIL (rhApo2L/TRAIL), a dual proapoptotic receptor agonist has been successfully studied in patients with advanced cancer in clinical trial for cancer therapy [20,21].

Our previous study showed that expression of TRAIL in cancer cells induced apoptosis and bystander effect and that the bystander effect required cell-cell contact antitumor activity [22,23]. Required cell to cell contact suggests that interaction of membrane TRAIL with its receptors (DR5 or DR4) in neighboring cells is an important mechanism of the TRAIL induced cell death. However, it remains unclear whether endogenous expression of TRAIL itself will be sufficient to induce cell death. In this study, we compared Ad/gTRAIL induced cell death in relative confluent cells and in very diluted cultured cells where cell-cell contact was minimal. Our result showed that treatment with Ad/gTRAIL virus elicited similar levels of apoptosis in the confluent and diluted cells, suggesting that endogenous expression of TRAIL itself is sufficient to induce cell death in the sensitive cancer cells (data not shown).

Control of gene expression via tissue-or cell-specific promoters is a means of targeting transgene expression. Tumor-specific promoters have the potential to selectively destroy cancer cells while sparing normal cells. We previously showed that hTERT promoter is highly active in most cancer cells but not in normal cells and normal mouse tissues, and can be used for targeting of the therapeutic effects of a proapoptotic gene using adenoviral vector mediated gene delivery [12,24]. Nevertheless, comparing with a strong constitutive promoter such as CMV promoter, the activity of hTERT is relatively weak. Therefore, Ad/gTRAIL used bicistron expression system to augment the hTERT promoter activity in cancer cells. In this bicistron system, hTERT promoter is used to drive the expression of GAL4/VP16 which in turn activates its target promoter consisting of GAL4 binding sites and TATA box (GT)[25]. Targeted expression of green Antitumor activity leading to augmented transgene expression. Our previous study showed this system could augment transgene expression from the CEA or hTERT promoter up to 100 fold, whether in vitro or in vivo without loss of its specificity [26,27].

Recombinant TRAIL proteins could elicit anticancer activity without significant toxicity to most normal cells [28]. Direct introduction of the TRAIL gene into cancer cells elicited apoptosis and suppress tumor growth in vitro and in vitro. A notable advantage of TRAIL gene therapy is that TRAIL can kill nontransduced neighboring cancer cells through bystander effects. TRAIL may become a therapeutic agent for certain cancers. However, recent findings that normal human hepatocytes, brain tissue, and certain epithelial cells are susceptible to recombinant TRAIL proteins[29], and that nonselective expression of TRAIL gene in mice caused serious liver toxicity raised concerns about the potential toxicity of systemically administration of TRAIL. Targeting TRAIL gene expression to cancer cells by hTERT is expected to improve the safety of TRAIL gene therapy. Previous study showed that treatment of some lung and colon cancer cells with Ad/gTRAIL elicited strong antitumor activity *in vitro *and *in vivo *without toxic effects on primary human hepatocytes and in mice [25]. In breast cancer cells, Ad/gTRAIL was effective against cell lines resistant to doxorubicin and soluble TRAIL protein. Intralesional administration of Ad/gTRAIL effectively suppressed xenografted tumor growth resulted in long-term tumor-free survival in half of the mice [30]. This data suggests that Ad/gTRAIL has potential application in cancer therapy.

Meningiomas and gliomas are among the best candidates for intralesional treatment with gene based agents [31]. In addition, malignant gliomas and meningiomas are predominantly telomerase positive, while normal brain tissues are telomerase negative. Kondo and his colleagues made a couple of plasmid constructs in which various caspases were put under the control of hTERT promoter and they used these constructs for in vitro and in vivo anti-glioma studies via liposome delivery system. They showed that hTERT/caspase constructs induced apoptosis in hTERT-positive malignant glioma cells but not in astrocytes or fibroblasts in vitro [3,18]. Moreover, these constructs significantly inhibited subcutaneously established meningioma and glioma tumors in nude mice by seven daily intratumoral injections. These studies provided the proof-of-principle evidence that hTERT promoter-driven suicide therapy may become a novel approach for the treatment of malignant meningiomas and gliomas. Although *in vitro *and *in vivo *results are encouraging, the potential use of these plasmid constructs in clinical settings is less promising. Compared with other vector system, especially adenovirus, the expression level mediated by liposome-DNA delivery is significantly lower when intratumoral injection is performed, whereas systemic treatment of malignant brain tumor by gene therapy may not be possible clinically.

Adenovirus is the most widely used vector in cancer gene therapy. Numerous clinical trials are based on adenoviral vector system [32,33]. We have made several adenoviral constructs using hTERT promoter to drive various apoptotic genes. Ad/gTRAIL is one of these constructs. In this study, we applied Ad/gTRAIL vector to glioma cells with varying malignancies. We found that the response of meningioma and glioma cells to Ad/gTRAIL treatment correlate with their malignancy level. Those with high malignancies are susceptible to Ad/gTRAIL treatment, while benign lower grade glioma cells and benign meningioma cells have very low hTERT expression and hence low GFP-TRAIL expression. Ad/gTRAIL may become an effective therapeutic agent for malignant brain tumors in cases where surgery is not an option and even as a prophylactic agent to prevent recurrence.

## Methods

### Primary cell culture and cell lines

Human tumor specimens were obtained and stored in accordance with the human subject research protocols approved by the institutional review board. The processing of tumor specimens has been described previously [32,33]. Briefly, after resection, a portion of each tumor was sent for routine histopathological analysis. The remainder of all samples was immediately used to establish primary cultures. Tumor fragments were dispersed into individual cells by treatment with Dispase 1 for 15 to 30 min at 37°C. From each tumor, 1 × 10^6 ^cells were then plated in a 100 mm tissue culture dish in low-glucose Dulbecco's modified Eagle's medium (DMEM) with 10% fetal bovine serum (FBS), and 1% penicillin/streptomycin mixture. The cells were grown to confluence and then harvested, aliquoted, and stored in liquid nitrogen for future use. The U251, U87, U373, and SW1088 glioma cell lines were obtained from American Type Culture Collection (Manassas, VA). The NG-1 glioma cell line was a gift from Dr. TF Liu (University of Texas MD Anderson Cancer Center). U251, U87, U373, SW1088 and NG-1 glioma cell lines were grown in DMEM high glucose, L-glutamine medium with 10% FBS and 1% penicillin/streptomycin mixture. All cells were cultured in a humidified atmosphere containing 5% CO_2 _at 37°C.

### Recombinant adenovirus vectors

Ad/gTRAIL were constructed as described previously[34,35]. Ad/CMV-GFP was provided by Dr. T.J. Liu in our institution. Virus titers were determined by optical absorbance at A260 and by plaque assay. Particle/plaque ratios normally fell between 30:1 and 100:1. Based on a report by others on evaluation of the concentration [36], and our own experience, vector titers determined by A260 were used in this study while titers determined by plaque assays were used as additive information. Thus, the multiplicity of infection (MOI) of 1000 VPs was equivalent to an MOI of 10-30 infectious units. Unless otherwise specified, Ad/CMV-GFP was used as the vector control, and PBS was used as a mock control. All viral preparations were free of contamination by E1^+ ^adenovirus and endotoxin.

### Adenovirus infection

For the infectivity analyses, human benign and malignant tumor cells (5 × 10^5 ^) were infected with 1000 MOI Ad/g-TRAIL, 1000 MOI Ad/CMV-GFP, or PBS, in which Ad/g-TRAIL, Ad/CMV-GFP-infected cells express GFP. 72 hours after infection, the cells were treated with 0.05% trypsin for 5 minutes and washed twice with phosphate-buffered saline (PBS). The cells were then counted for GFP-positive cells by flow cytometry as described below, or visualized and photographed by using a Nikon Eclipse TE300 inverted fluorescence microscope (Nikon, Melville, NY) and were analyzed with MetaMorph imaging software (Universal Imaging Corp Downington, PA).

### Cell viability assay

Human benign and malignant tumor cells were seeded in at a density of 3 × 10^3 ^cells/well in 96 well plates and allowed to grow for 20 hours at 37°C. Cells were then infected with 1000 MOI Ad/g-TRAIL, 1000 MOI Ad/CMV-GFP, or PBS at 7 days respectively. Cell viability was determined by 3-bis-[2-methoxy-4-nitro-5 sulfenyl]-2*H*-tetrazolium-5-carboxanilide inner salt (XTT) assay (Cell Proliferation Kit II; Roche Molecular Biochemicals, Indianapolis, IN) following manafacturer's instructions. Each experiment was performed in quadruplicate and repeated at least three times.

### Real-time quantitative RT-PCR

Total RNA was extracted using the Mini-prep RNeasy kit (Qiagen). cDNA synthesis was constructed from high-grade RNA from all samples using the High Capacity cDNA Reverse Transcription kit (Applied Biosystems, Foster City, CA, USA). Real-time Q PCR was performed in the ABI Prism 7700 Sequence Detection System according to the protocol of the manufacture. Typical amplification mixes (25μl) contained the sample DNA (or cDNA), 10× TaqMan Buffer (2.5μl), 200μm dATP, dcP, dGTP, and 400μM dUTP, 5 mM MgCl_2_, 0.65 units of Ampli Taq Gold, 0.25 units of AmpErase uracil N-glycosyladse (UNG), 200 nM each primer and 100 nM probe. The thermal cycling conditions consist of 1 cycle at 2 min for 50°C and 10 min for 95°C, and 50 cycles of 95°C 15 s and 60°C for 1 min. All reactions were performed in duplicates. After the reaction, we used the built-in software in the 7700 system to perform analyses of the data and generate the standard curve, the Ct value of each testing sample and their corresponding starting quantity based on the relative standard curve.

### Western blot analysis

The cells treated with 1000 MOI Ad/CMV-GFP or 1000 MOI Ad/g TAIL at 3 days, the cell protein extraction was performed with Laemmli lysis buffer. Equal amounts of lysate were separated using 10% SDS-PAGE and transferred to Hybond enhanced chemiluminescence membranes (Amersham, Piscataway, NJ). The membranes were blocked with PBS-T containing 5% non-fat milk for 1 h or overnight at 4°C, and incubated with primary antibodies for 1 h at room temperature. After washing three times with PBS containing 0.05% Tween, the membranes were incubated with peroxidase-conjugated secondary antibodies and developed using a chemiluminescence detection kit (ECL kit; Amersham).

### Fluorescence-activated cell sorting (FACS) and flow cytometric analysis

Cells were seeded at 1 × 10^5^/well in 6-well plates, and after an overnight incubation, cells were either treated with 1000 MOI Ad/CMV-GFP or Ad/gTRAIL or complete media (control cells) for 24 hours. 72 hours later, both adherent and floating cells were harvested by trypsinization, washed with PBS, and fixed in 70% ethanol overnight at 4°C. Before analysis, cells were stained with propidium iodide for 30 min. The apoptosis induction was quantified by flow cytometric analysis. All experiments were performed in the Core Laboratory of the M. D. Anderson Cancer Center.

### Intracranial xenografting of human meninglioma and glioma cells

#### Animals

Protocol for animal use was approved by the Institutional Animal Care and Use Committee of Baylor College of Medicine, and was in accordance with National Institutes of Health guidelines (NIH publication number 85-23).

#### Procedure

A total of 24 female, 10-weeks-old, *nu/nu *athymic mice (Charles River Lab) were used. Human meningioma M6 and glioma U87 cell lines (at a concentration of 1 × 10^6 ^cells/5 μL) were resuspended in PBS and injected into the right frontal lobe of nude mice using a guide-screw system implanted within the skull as described previous [21,37,38]. On day 3, after the implantation of tumor cells, animals were divided into four groups, and the each group is treated with one single intratumoral injection (1.5 × 10^8 ^viral particles in 5 μL) with following: the first group animals (n = 6) were treated with Vectors Ad/CMV-GFP for meningioma M6; the second group animals (n = 6) were treated Vectors Ad/gTRAIL for meningioma M6; the third group animals (n = 6) were treated with Vectors Ad/CMV-GFP for glioma U87; the fourth group animals (n = 6) were treated with Ad/gTRAIL for glioma U87. Mice were anesthetized with xylazine/ketamine during the procedure. Mice showing general or local symptoms of toxicity were killed. When the animals became moribund due to tumor progression, they were euthanized and the brains were removed, fixed in 4% formaldehyde for 24 h at room temperature

### Histology and tumor analysis

Brain tissue fixed for 24 h in 10% formalin solution, transferred to a 70% ethanol solution, and processed for paraffin embedding. Serial sections (6 μm) were prepared and stained with H&E according to standard histopathologic techniques. Stained sections were examined under light microscope (×100 magnification).

### Apoptosis assay

To detect apoptotic cells in tumor, we used an in situ cell death detection kit, POD (Roche Applied Science, Indianapolis, IN). The staining was performed according to the manufacturer's instructions, counterstained with haematoxylin, and viewed under a light microscope (×400 magnification). Brown staining indicates oligonucleosome cytoplasmic release resulting from apoptosis-induced DNA fragmentation. Counting was performed in randomly chosen fields, and the apoptosis was calculated as a percentage of at least 1,000 scored cells. Data analyzed with ImageJ software. Images segmented and the number of apoptotic cells quantified.

The standard fluorescein isothiocyanate-dependent apoptosis assay techniques (TUNEL or Annexin V) could not be used in this study because Ad/gTRAIL-infected cells express GFP, which interferes with fluorescein isothiocyanate detection by means of flow cytometry.

### Statistical analysis

Differences among the experimental groups were analyzed by analysis of variance (AOV) using statistical software (StatSoft, Tulsa, OK). A difference was considered statistically significant when the *P *value was 0.05 or less. Differences in tumor growth *in vivo *among the treatment groups were assessed by AOV with a repeated measurement module. AOV was performed to determine statistical significance between each treatment group by using the SAS procedure with the SAS version 6.12 software. Survival was assessed by using the Kaplan-Meier method. Survival in different treatment groups was compared using the log-rank test.

## Abbreviations

**hTERT**: Human telomerase reverse transcriptase; **TRAIL**: Tumor necrosis factor related apoptosis-inducing ligand; **GFP**: Green fluorescent protein; **A/gTRAIL**: Adenoviral vector with human telomerase reverse transcriptase promoter driven tumor necrosis factor related apoptosis-inducing ligand fusion gene; **Ad/CMV**: Adenoviral vector with Cytomegalovirus promoter; **FACS**: Fluorescence-activated cell sorting; **TUNEL: **Terminal deoxynucleotidyl transferase- dUTP nick end labeling; **dUTP**: 2'-Deoxyuridine, 5'-Triphosphate; **DR**: death receptors; **DISC**: Death-inducing signaling complex **Apo2L/TRAIL**: (rhApo2L/TRAIL)- proapoptotic receptor agonist; **DMEM**: Dulbecco's modified Eagle's medium; **FBS**: Fetal bovine serum; **XTT**: 3-bis-[2-methoxy-4-nitro-5 sulfenyl]-2H-tetrazolium-5-carboxanilide inner salt; **NG-1**: neural glioma **MOI-: **Multiplicity Of Infection; **SDS-PAGE**:Sodium dodecyl sulfate polyacrylamide gel electrophoresis; **PBS**: phosphate-buffered saline; **UNG**: Uracil N-glycosyladse **RT Q-PCR**: Real-time quantitative polymerase chain reaction; **AOV**: Analysis of variance

## Competing interests

The authors declare that they have no competing interests.

## Authors' contributions

JL, IM and BF designed the study. JL draft of the manuscript, JL, AZ, performed experiments, WA, BF, participated in manuscript correction and Performed data analysis, KB RN, DK, FM, IM revised the manuscript. All authors read and approved the final manuscript.
